# Professor Flint

**Published:** 1853-07

**Authors:** 


PROFESSOR FLINT.

On receiving from Professor Flint his letter of resignation, the Council of the University of Buffalo passed the following complimentary resolutions:

"1. *Resolved*, That the Council accept, with sincere sorrow, the resignation of Dr. Flint of the important Chair so long and ably filled by him.

2. *Resolved*, That in his whole connection with the University, Dr. Flint has labored with untiring zeal for its interests, and with distinguished success in their attainment. That connection dates from the earliest inception of the school; and in all the vicissitudes which have attended its existence, Prof. Flint has ever been foremost in sustaining in this city the cause of sound medical education. And the Council, while they attribute to the talents of Dr. Flint no small share in the success of the institution, are rejoiced to know that, in thus building up and sustaining it, he has found his reward in a wide-spread reputation as one of the ablest teachers of practical medicine in our country.

3. *Resolved*. That in severing the relations that have united us so long, we tender to Dr. Flint our hearty thanks for his valuable services, and our earnest wishes that in the University of Louisville, to which he has now been called, he may find a pleasant and ever enlarging field of professional labor."

